# Physicians’ Perceptions of Clinical Decision Support to Treat Patients With Heart Failure in the ED

**DOI:** 10.1001/jamanetworkopen.2023.44393

**Published:** 2023-11-21

**Authors:** Scott D. Casey, Mary E. Reed, Chris LeMaster, Dustin G. Mark, Jesse Gaskin, Robert P. Norris, Dana R. Sax

**Affiliations:** 1 Kaiser Permanente Division of Research, Oakland, California; 2 The Kaiser Permanente CREST Network, Oakland, California; 3 The Permanente Medical Group, Oakland, California; 4 The Permanente Medical Group Consulting Services, The Permanente Medical Group, Oakland, California; 5 The Permanente Medical Group, Sacramento, California

## Abstract

**Importance:**

Clinical decision support (CDS) could help emergency department (ED) physicians treat patients with heart failure (HF) by estimating risk, collating relevant history, and assisting with medication prescribing if physicians’ perspectives inform its design and implementation.

**Objective:**

To evaluate CDS usability and workflow integration in the hands of ED physician end users who use it in clinical practice.

**Design, Setting, and Participants:**

This mixed-methods qualitative study administered semistructured interviews to ED physicians from 2 community EDs of Kaiser Permanente Northern California in 2023. The interview guide, based on the Usability Heuristics for User Interface Design and the Sociotechnical Environment models, yielded themes used to construct an electronic survey instrument sent to all ED physicians.

**Main Outcomes and Measures:**

Main outcomes were physicians’ perceptions of using CDS to complement clinical decision-making, usability, and integration into ED clinical workflow.

**Results:**

Seven key informant physicians (5 [71.4%] female, median [IQR] 15.0 [9.5-15.0] years in practice) were interviewed and survey responses from 51 physicians (23 [45.1%] female, median [IQR] 14.0 [9.5-17.0] years in practice) were received from EDs piloting the CDS intervention. Response rate was 67.1% (51 of 76). Physicians suggested changes to CDS accessibility, functionality, and workflow integration. Most agreed that CDS would improve patient care and fewer than half of physicians expressed hesitation about their capacity to consistently comply with its recommendations, citing workload concerns. Physicians preferred a passive prompt that encouraged, but did not mandate, interaction with the CDS.

**Conclusions and Relevance:**

In this qualitative study of physicians who were using a novel CDS intervention to assist with ED management of patients with acute HF, several opportunities were identified to improve usability as well as several key barriers and facilitators to CDS implementation.

## Key Points

**Question:**

What are physicians’ perceptions of using a novel clinical decision support intervention that displays risk estimates, collates relevant clinical information, and provides treatment recommendations for patients with acute heart failure in the emergency department?

**Findings:**

In this qualitative study with 58 emergency department physicians, participants identified several actionable items that they believed would enhance clinical decision support usability and workflow integration. Most physicians believed that clinical decision support would improve emergency department treatment of patients with heart failure as well as patient outcomes.

**Meaning:**

Emergency department physicians found value in a novel clinical decision support intervention providing risk estimates, clinical information, and treatment recommendations for patients with acute heart failure and proposed several key enhancements that may facilitate its implementation and benefits.

## Teaser

This qualitative study evaluates clinical decision support usability and workflow integration among emergency department (ED) physicians who use it in clinical practice.

## Introduction

### Background

Incorporating prediction models and decision support interventions in a busy emergency department (ED) is challenging^1,2,3,4^ and requires a thorough understanding of threats to usability, implementation, and physician stakeholder acceptance.^1,2,5,6,7,8^ Furthermore, understanding stakeholders’ perceptions of using technology as an adjunct to clinical decision-making is especially important when implementing interventions designed for high-risk conditions such as acute heart failure (HF). Several recent studies document unsuccessful attempts to incorporate electronic health record (EHR)-integrated clinical decision rules when these interventions were deployed without careful attention to user needs and existing workflows.^9,10,11,12^ The REVEAL-HF trial^13^ implemented an EHR alert displaying 1-year HF mortality risk that failed to change physician medical decision-making or patient outcomes. REVEAL-HF authors cited the intervention’s lack of specific, actionable recommendations to explain this finding.^13^

Patients with HF are a socially and medically complex population. Social determinants of health disparities such as unemployment, education, and disability appear to have a disproportionate effect on patients with HF.^14^ These patients have frequent ED visits where a high percentage are hospitalized.^15^ Stratifying ED patients with acute HF according to short-term risk of serious adverse events (death, cardiopulmonary resuscitation, balloon-pump insertion, intubation, new hemodialysis requirement, myocardial infarction, or coronary revascularization) is challenging, and may contribute to both high hospital admission rates as well as high rates of adverse events among patients discharged from the ED.^16,17^ To address this misalignment of care and improve patient outcomes, clinical decision support (CDS) interventions have been developed to risk-stratify ED patients with HF and assist with disposition decision-making.^16,18,19,20^ CDS encompasses a range of technological interventions providing patient-specific information at appropriate times to health care clinicians and includes computerized alerts, patient care reminders, and disease-specific order sets.^21^ Risk prediction models coupled to CDS may improve disposition decision-making for ED patients with acute HF and are recommended by the American College of Emergency Physicians’ clinical policy on heart failure syndromes.^22,23,24^

Our study team recently developed an acute HF risk prediction model incorporating more than 60 variables that estimates risk of 30-day serious adverse events.^16^ We built the risk model and corresponding CDS into the EHR and conducted a pilot study in 2 EDs within our large, integrated health care delivery system from January 2023 through March 2023.

Physician-facing CDS powered by risk prediction models can improve ED care of complex, high-risk patients. A thorough understanding of the environment in which the CDS will be used as well as threats to CDS usability is crucial to its implementation and sustainability.

Our objective was to assess CDS usability as well as barriers and facilitators to its workflow integration and widespread implementation by understanding the perspectives of ED physician end users who use the intervention to assist them in managing patients with acute HF.

## Methods

### Study Design and Setting

We used a mixed-methods qualitative study design to gather quantitative and qualitative feedback from a group of community ED physicians from 2 medical centers of Kaiser Permanente Northern California (KPNC) participating in a pilot study of the risk prediction model and CDS. KPNC is an integrated health care delivery system with 21 hospital-based medical centers serving nearly 5 million members whose demographic characteristics are reflective of the regional population.^25^ Compared with other hospitals across the United States, KPNC hospitalizes a relatively lower percentage (57%) of ED patients with acute HF, with 30-day mortality rates among discharged ED patients comparable with those reported in other settings.^16,26,27,28^ The 2 EDs are located in urban areas and function as a single medical center with a shared physician group and specialty service availability. The combined volume between the 2 centers is approximately 120 000 patients per year and the hospital admission rate is approximately 12%. Both facilities use the Epic EHR system and have resident and medical student learners rotating in the ED. One of the facilities has a catheterization laboratory and an ED observation unit, and patients requiring these services in the ED that lacks these resources are transferred to this facility.

Physicians in our study participated in 1-on-1 usability sessions and semistructured interviews, which informed the design of an electronic survey instrument sent to all ED physicians at the study sites. The survey included ordinal, categorical, and free-text responses. All full-time physicians in the group received an email invitation to complete the survey and were offered a $75 gift card for their participation. The KPNC institutional review board approved the study with a waiver of the requirement to obtain informed consent for the data-only portion of the study and with a waiver of documentation of informed consent (no signature required) for physician interviews. This qualitative study followed the Consolidated Criteria for Reporting Qualitative Research (COREQ) reporting guideline.^29,30^

### Study Population and Selection

A convenience sample of physicians who worked at the 2 EDs involved in our CDS pilot study were selected to participate in our usability studies and semistructured interviews. During the pilot study, there were 76 full-time physicians covering the 2 included EDs. All full-time physicians at these EDs were invited to participate in usability interviews by email and by announcements made at monthly department meetings. All full-time ED physicians were sent an electronic link to the survey and were reminded about participation in 3 subsequent emails.

### Clinical Decision Support Tool

We previously described the design and performance characteristics of a risk prediction model in estimating 30-day serious adverse events.^16^ The model incorporates more than 60 clinical, laboratory, and sociodemographic variables.^16^ We previously demonstrated excellent discrimination using logistic regression-based (area under the curve [AUC], 0.80 [95% CI, 0.79-0.82]) and machine learning-based (AUC, 0.85 [95% CI, 0.83-0.86]) approaches.

We coupled the risk prediction model to an EHR-embedded CDS informed by physicians’ preferences (eFigure 1 in Supplement 1).^31^ We envisioned the CDS to serve several functions: (1) collate relevant patient-specific clinical information in a single location (eg, recent cardiac studies, laboratory values, weights, vital signs, active cardiac medications); (2) provide recommendations on medical management in the ED including diuretic strategies and specific recommendations to enhance adherence to Guideline Directed Medical Therapy (GDMT)^32^; and (3) display patients’ 30-day risk of serious adverse event and corresponding ED disposition recommendation.^31^ We chose a passive physician prompt to encourage, but not mandate, interaction with the CDS, based upon initial physician surveys.^31^

ED physicians in the EDs who participated in the CDS pilot study received education on CDS use which consisted of two 30-minute virtual and one 30-minute in-person training sessions as well as monthly email communications. Patients meeting eligibility criteria (based on an algorithm that included history of HF, relevant chief complaint, and placement of HF-relevant orders) during the pilot study were flagged in the EHR via a passive prompt located on the top of 1 screen that ED physicians may use to view relevant clinical information in the EHR.

### Research Team and Reflexivity

Three practicing ED physicians (S.D.C., D.R.S., C.H.L.) and an expert in human-centered design strategy (J.G.) conducted the usability study and semistructured interviews. Of these, 2 physicians (D.R.S., C.H.L.) had professional relationships with participants and 1 (D.R.S.) had previous experience with qualitative studies. The remaining team members (S.D.C. and J.G.) had no professional relationships with participants although all were employed by the same health system.

### Data Collection

Usability sessions were approximately 60 minutes in length and conducted in March 2023. Interviews were conducted either in a hospital conference room or at an investigator’s home. We used an interview guide (eFigure 2 in Supplement 1) that was piloted by 3 ED physicians (S.D.C., D.R.S., C.L.). We conducted usability studies using a deductive dominant, “think aloud” approach to “near live” clinical scenarios^33,34^ allowing for emergent themes (inductive approach) to develop. After obtaining verbal consent, investigators explained that the aim of the study was to understand physicians’ interactions with the EHR during the care of ED patients with acute HF and their perspectives on using the CDS and to identify perceived barriers and opportunities to CDS adoption. We collected demographic information such as physician-specified gender, number of years in practice, and number of years at current position. We recorded field notes during interviews and captured video and transcribed text using Microsoft Teams (Microsoft Corp).

For the usability testing session, participants were asked to review triage vital signs, mode of arrival, and chief concern of a sample patient with HF on the ED trackboard within the EHR. They were asked to “think aloud” and verbalize their current approach to reviewing relevant medical data, placing orders, assigning risk, and deciding ED disposition. If the participant did not access the CDS on their own, a member of the study team asked the participant to locate the CDS and verbalize how they might use it to assist with clinical decision-making. Participants were then asked additional questions as part of a semistructured interview probing the following sociotechnical environment domains: (1) internal policies, (2) human-computer interface, (3) workflow, (4) people, and (5) clinical content.

### Data Analysis

We identified usability themes from our physician interviews using content analysis based upon a model incorporating heuristics for user interface design^35^ and thematic analysis to identify themes based upon the 8-dimensional Sociotechnical Framework for assessing design, implementation, and use of health information technology.^36^ Two team members (S.D.C. and D.R.S.) independently reviewed transcripts to create an initial coding framework using comparison and consensus before independently coded transcripts. The principal investigator reviewed all transcripts for coding discrepancies and these were discussed with the study team. The codebook was collaboratively reviewed and refined as new codes emerged or existing codes were clarified (eFigure 3 in Supplement 1). Interviews were conducted until information power was reached.^37^ The qualitative data management software Dedoose version 9.0.62 (Dedoose) was used for coding and analysis of transcripts. We used the methods of Lincoln and Guba to ensure qualitative rigor (eTable in Supplement 1).^38^ Survey data was presented graphically using Microsoft Excel version 2105 (Microsoft Corp). Data analysis was performed from May 1 to June 30, 2023. Participants were offered a $75 gift card for their participation and a copy of their interview transcript for future feedback.

Themes identified from our physician interviews informed the design of an electronic survey instrument which elicited categorical, ordinal, and Likert scale responses. The survey instrument was pretested by 4 practicing ED physicians and iterative changes were made to clarify the instrument prior to inviting participation from all full-time ED physicians who staff the 2 EDs participating in the CDS pilot study.

## Results

After conducting 7 interviews (mean [range] duration, 50 [35-63] minutes) with physicians (5 [71.4%] female; median [IQR] 15.0 [9.5-15.0] years in practice) working in the 2 EDs that piloted the CDS, we reached sufficient information power.^37^ Physician usability sessions and semistructured interviews yielded several themes along with representative quotations that illustrated several key domains of the Sociotechnical Environment and CDS usability frameworks (Table 1 and Table 2; eFigure 4 in Supplement 1). We invited 76 physicians to participate in our electronic survey which was returned by 51 physicians (67.1% response rate) (Table 3). Among these 51 physicians, 36 physicians (70.6%) preferred being able to voluntarily access the CDS from additional locations, and 23 (45.1%) were amenable to an involuntary opt-out approach to CDS prompting (Figure); 48 physicians (94.1%) agreed that the CDS would improve patient outcomes and 31 (60.8%) agreed it would save time. There were 31 physicians (60.8%) who reported using the CDS during the 3-month pilot and fewer than half (21 [41.2%]) agreed that the CDS was easily located. Most physicians (36 [70.6%]) expressed a preference for being able to access the CDS from multiple locations within the EHR. During the CDS pilot study, there were 703 patients (median [IQR] age, 76 [66-84] years; 374 [53.2%] female, 214 [30.4%] White, 260 [37.0%] Black, 103 [14.7%] Hispanic, 121 [17.2%] Asian) that met criteria to generate a CDS prompt to ED physicians.

**Table 1.** Themes Describing Facilitators to CDS Implementation Derived From Emergency Department Physician Interviews

Domain or theme	Representative quote (participant)
Clinical content	
Useful recommendations	“…These medications are really important as we’re getting more familiarity with the medications that patients should be on….I knew this sort of theoretically, but it’s great to see that [medication recommendations] here.” (Participant 5)
	“Right now, I’m probably not doing anything other than telling them to double up on their Lasix and call their primary care doctor. So, I think that it [CDS recommendations] would make me more likely to prescribe new medications to these patients.” (Participant 4)
	“I would definitely use it [CDS] for deciding on a tight discharge bundle for them [patients]…looking at what [medications] they’re on and then what the recommendations are.” (Participant 2)
Quantitative risk assessment	“There’s something so concrete about it [quantitative risk provided by the CDS] that I think people end up feeling reassured.” (Participant 3)
	“…if you say the PORT score or the PESI score…it’s a data point and a conversation between colleagues.” (Participant 4)
Efficiency	“I think that’s exactly the stuff that I want…I want echos (echocardiography)…I want caths [cardiac catheterization data], I want relevant medications…I want to know if they’ve had a stress test….” (Participant 2)
Workflow	
Complements workflow	“It [a CDS recommendation to admit a high-risk patient] certainly would nudge me in that direction if I was already on the fence and the patient came up as high risk.” (Participant 5)
	“…If I see this pop up with ‘high risk’ in a patient that I’m considering discharging, it would make me think twice about it.” (Participant 2)
	“Maybe if I was discharging a patient home, I would route my note to the primary care physician and I would say ‘This patient, based on our heart failure risk tool, had a really high risk of mortality in X days...is there any way that you can see this patient within the next week or so?’” (Participant 6)
	“I think it’s [CDS] going to help with giving me information quickly. I think it’s going to help with disposition decision-making and with being able to provide concrete information to the admitting hospitalist….” (Participant 1)
	“…at least 1 time I remember that it [the tool] did not change my eventual decision, but it did change the thoroughness with which I spoke to the patient and made a plan….” (Participant 2)
People	
Trust	“…because of where it [CDS and research used to develop it] came from, I don’t need more information. I don’t have the data analysis background…but I’m comfortable asking [others] to help me.” (Participant 4)
Human-computer interface	
Efficiency	“I think it can complement it [workflow] because I feel like the information is so scattered within the I that it’s a more efficient use of my time if it’s just curated in one spot.” (Participant 6)
	“It’s going to help me filter my clinical impression a little bit more elegantly…then I already have an idea of which way they’re going so it helps with the timeline…if I know they’re going to be admitted I can make that happen faster.” (Participant 1)

Abbreviations: CDS, clinical decision support; I, electronic health record; PESI, pulmonary embolism severity index; PORT, pneumonia severity index.

**Table 2.** Themes Describing Barriers to CDS Implementation Derived From ED Physician Interviews

Barriers to CDS use	Representative quote (participant)
Human-computer interface	
Dyssynchronous use	“…a banner alert that you would get when you open up the patient for the first time…because otherwise I’m going to go immediately into my usual thing….” (Participant 1)
Aesthetic	“…[clickable drop-down menus] would be less psychologically intimidating.” (Participant 2)
	“This doesn’t really jump out at you. The orange color doesn’t. Maybe if it was a brighter orange.” (Participant 7)
Information saturation	“It’s too long. It’s just not going to happen. Nobody’s going to start these things or read through the end of this.” (Participant 1)
	“I think this tool actually has a lot of the information [in 1 place] that I was gathering [elsewhere] so I think I would be tempted to use this….” (Participant 2)
Medication ordering	“I’d love to be able to have this [CDS] up while I order so that I can look at the cardiac meds that are already there and compare that to what the recommendation is while I’m ordering.” (Participant 1)
	“I would love to see those 2 things [GDMT recommendations and medication ordering panel] side-by-side, then I don’t have to remember and go back.” (Participant 2)
Internal policies	
Scope and responsibility boundaries	“Wow, so we are supposed to start all of these things…but this is not realistic to have the average ER doctor do this….” (Participant 1)
	“I’m not meaning to be work averse, but that sounds like the job of the cardiologist to put in palliative care referrals for their own patients.” (Participant 4)
Clinical content	
Quantitative risk assessment	“…that’s a very hard thing to tell a patient [they have a high 30-day risk estimate]…I just met you and you need to come into the hospital and you have an X percent chance of dying in the next month….I don’t see anybody actually doing that.” (Participant 1)

Abbreviations: CDS, clinical decision support; ER, emergency room; GDMT, goal-directed medical therapy.

**Table 3.** Characteristics of Survey Respondents and Usability Testing Participants

Physician characteristics	No. (%)	
	Survey respondents (n = 51)	Usability testing participants (n = 7)
Years in practice, median (IQR)	14.0 (9.5-17.0)	15.0 (9.5-15.0)
Years in current hospital, median (IQR)	11.0 (7.0-16.8)	10.0 (9.0-13.0)
Age, y		
<35	1 (2.0)	0
36-45	21 (41.2)	3 (42.9)
46-55	29 (56.7)	4 (57.1)
Gender		
Women^a^	23 (45.1)	5 (71.4)
Men^a^	28 (54.9)	2 (28.6)
Used CDS ≥1 time	31 (60.8)	7 (100.0)

Abbreviation: CDS, clinical decision support.

^a^ Self-reported.

**Figure.**
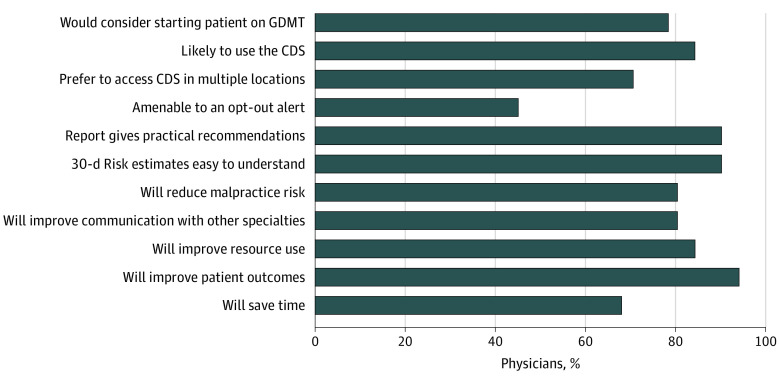
Percentage of Physician Survey Respondents Who Agree Or Strongly Agree With Survey Questions CDS indicates clinical decision support; GDMT, goal-directed medical therapy.

### Usability Domains

The Usability and Sociotechnical Environment models overlapped considerably at the human-computer interface domain. Physicians valued user control in their typical ED workflow allowing them to review clinical information, access results, and place orders at disparate times during an encounter. Some physicians felt that the collated clinical data displayed by the CDS was useful early in the visit, some felt it was useful for diagnosis and treatment recommendations (initial diuretic orders, interpretation of laboratory results), and some found that accessing the tool would be most helpful after initial ED treatment (site-of-care decision-making, shared decision-making, mortality estimates, and outpatient medication adjustments for patients safe for discharge). Despite this dyssynchronous use, all physicians expressed that they would be more likely to access the CDS if they were prompted early in the ED visit by a passive prompt encouraging, but not mandating, CDS interaction. Noted physician dissatisfaction with finding the CDS prompt and the limited ways to access it in the EHR represents an opportunity to improve usability.

Although most physicians appreciated having all pertinent HF data collated into a central location, most agreed that more customizability regarding how data was displayed would be helpful. Specifically, physicians requested an interface that would allow them to expand or collapse available data (ie, drop down lists) and organize clinical content in descending order of relevance. Additionally, many physicians wanted changes to the aesthetic design of the CDS recommending bolder colors to increase tool visibility and more succinct text to minimize information saturation.

Physicians noted that the need for information recall would be minimized if they were able to translate medication recommendations into orders directly from the CDS or at least access the order entry field while the CDS window was open (cannot order medications). Physicians also noted how the CDS risk estimates could result in faster disposition decision-making for HF patients.

### Sociotechnical Environment Domains

#### Internal Policies

All interviewed physicians noted that adherence to CDS recommendations would result in expansion of their current role in HF management. Several physicians noted that GDMT recommendations^32^ along with required outpatient laboratory monitoring (eg, creatinine, potassium) for discharged patients would add complexity to the ED discharge process. Some noted that this role could be carried out by other clinicians (ie, pharmacists, cardiologists, or primary care providers) not based in the ED.

Some interviewed and surveyed physicians reported that adherence to CDS recommendations would require extra clinical tasks (medication initiation, patient education, discussions with patients/caregivers on prognosis and goals of care) and noted that capacity constraints may prevent them from carrying out these new responsibilities during busy ED shifts. One physician noted that initiating a new GDMT medication in the ED was unrealistic and viewed this as a barrier to widespread CDS uptake. However, others appreciated the tailored medication recommendations suggested by the CDS and viewed them as an opportunity to improve patient outcomes. Most surveyed physicians indicated that they would consider starting GDMT medications for patients discharging from the ED.

#### Workflow

Many interviewed physicians reported that they consider multiple data points from different parts of their clinical workflow and evaluation to inform their decision to hospitalize a patient with HF, including current weight, vital signs, work of breathing, degree of response to ED treatment, degree of disability and social factors, and outcomes of shared decision-making conversations. Many interviewed physicians felt that the collation of information provided by the CDS would improve efficiency by presenting much of this important data. All physicians agreed that CDS risk estimates would be useful for disposition decision-making and to improve efficiency. Nearly all interviewed physicians reported that they would be very likely to reassess patients that they believed could be discharged but was labeled as “high risk” by CDS. Additionally, many reported that the risk estimates would affect how they structured their communication with the patient, their shared decision-making discussions, and their outpatient follow-up planning. Some interviewed physicians noted that CDS risk estimates would be useful in confirming that they had correctly identified a low-risk patient that they had selected for outpatient management.

#### People

Physicians showed willingness to adopt CDS-provided risk estimates into their clinical workflow and identified estimated risk to be an asset in conversations with patients and consultants. Concern regarding potential bias in machine learning models was identified by a few, but no physician reported that this would prevent them from using CDS. Physicians generally regarded machine learning models as a favorable health care innovation despite having limited understanding about how these models worked. Several physicians cited their trust in their local ED leadership in embracing technological innovations. Personal relationships with the study researchers were also reported to increase comfort with practice change.

#### Clinical Content

Multiple physicians reported that quantitative risk, a discrete number that could be communicated to patients and consultants, was helpful to the management of patients with HF. Physicians reported that CDS provided useful recommendations to help them translate this quantitative risk into appropriate management decisions.

## Discussion

Using a mixed-methods approach, we found broad support among physicians for the consolidated, HF-specific information and risk estimates provided by a CDS intervention. Physicians identified usability concerns as well as barriers and facilitators to CDS adoption and widespread use that informed iterative design.

We identified human-computer interface to hold the most opportunity for maximizing CDS adoption and continued use. Workflow integration, the temporal order that tasks are executed and the point in time in which the intervention will be used,^1^ was not consistent among physicians. Our usability testing sessions and survey responses suggested that physicians used (or would use) the CDS for multiple purposes during their workflow. We found that earlier prompting and CDS design changes that considered physicians’ variable interactions with the EHR might increase use. We identified that a main barrier to current CDS use was its placement in a single EHR location that was not viewed by most physicians. Furthermore, survey data indicated that physicians wanted the freedom to access the CDS from multiple locations in the EHR. A similar theme was identified in the BETTER CARE-HF trial,^39^ which placed prompts in several EHR locations (upon opening medical record, medication ordering, refill ordering) and found it to be associated with a doubling of GDMT compliance compared with usual care.

Physicians had other tangible and actionable recommendations to change the CDS prompt and CDS content that might increase adoption. These suggestions included changes in the visual display (color, quantity of text) as well as allowing for more autonomy over displayed CDS content. Our intervention used a passive prompt to encourage CDS interaction similar to the strategy used by BETTER CARE-HF. We chose this passive prompt to minimize alert fatigue, which has been reported to create a gradual desensitization to electronic alert messages with prolonged prompting.^40^ Adopting a mandatory opt-out approach may increase CDS use, however, it may contribute to alert fatigue which would hinder implementation and long-term sustainability.^40,41^ The PROMPT-HF trial recently demonstrated an association between an opt-out physician prompting strategy and increased GDMT prescribing compliance.^42^ Physicians were prompted to use CDS whenever they accessed the EHR’s order entry module for eligible patients with HF.^42^ However, PROMPT-HF’s population was ambulatory and cannot be directly compared with our own. Also, the trial’s brief study period likely precludes a true assessment of alert fatigue.^40^

Physicians noted that in its current iteration, the CDS was limited by the lack of ability to integrate recommendations seamlessly into clinical workflows. In its current form, physicians had to first exit the CDS, recall the specific medication and dose mentioned in the CDS, and then place the orders in a separate window, leading to excess cognitive burden and prolonged workflows.^1^ In the setting of multiple concurrent distractions in the ED work environment, clinicians noted that this was a substantial limitation to use.

Physicians identified concerns about their capacity to meet CDS expectations and cited these concerns as potential limitations to adoption. While physicians recognized that cues to adhere to GDMT prescribing represented an opportunity to improve HF patient care, they worried about their capacity to consistently follow these guidelines due to excess workload. Additionally, GDMT prescribing to patients being discharged home was viewed by some as falling outside of traditional ED practice and was more appropriately managed by other services (cardiology, primary care, specialty HF chronic care programs). These considerations are important to consider as interventions that increase physician task load pose psychological and behavioral barriers to the adoption ED interventions.^2,43^ Encouragingly, our physician survey results indicated that most physicians believed that CDS will improve guideline-concordant treatment and result in improved patient outcomes. Future physician education will be important for physicians to embrace the association between improved long-term clinical outcomes and GDMT prescribing.

Unfamiliarity and/or distrust regarding machine learning models is often discussed as a barrier to deploying these tools in the ED.^5,44^ However, physicians in our study did not identify use of machine learning methods as a barrier to CDS use. Personal relationships with fellow ED physicians who served as advocates of the intervention likely mitigated these concerns. Trust emerged as an important theme that contributed to physician readiness to incorporate CDS risk estimates into clinical decision-making despite limited understanding of the risk model’s origin. Physicians included in our study had prior access to EHR-embedded CDS for other ED conditions and this familiarity may have increased their willingness to adopt the HF CDS during the pilot study.

Physicians in our study found the CDS clinical content helpful for their management of patients with HF. Despite having a median of 15 years in practice and well-established practice patterns for patients with HF, physicians reported the clinical recommendations included in the CDS were (or could be) useful in their clinical practice. Although risk estimates provided by the CDS were a novel addition to their workflow, physicians indicated that they had value. Physicians readily recognized that CDS was applicable to both low-risk populations (reassurance of a safe discharge plan) and high-risk populations (shared decision-making, facilitated discussions with consultants, and more careful transitions of care for home patients) which may facilitate adoption and long-term use.

### CDS Redesign

In response to CDS usability results, we made several changes to improve accessibility, clinical content, and workflow. Passive prompts were placed in 2 additional locations in the EHR. We added additional HF-specific clinical data (most recent ejection fraction, patient weight trend, cardiology clinic notes) and reordered the presented data in order of relevance based on physician feedback. Additionally, we implemented individualized medication recommendations based upon patients’ current outpatient medication list to assist physicians in GDMT ordering. These new GDMT recommendations are presented as clickable text which linked physicians directly to the EHR ordering module. We also provided physicians with links to access consensus recommendations from the American Heart Association, European Society of Cardiology, and internal Kaiser Permanente clinical practice recommendations on GDMT.

Our mixed-methods approach assessed usability of a novel CDS intervention in the ED and identified barriers and opportunities to implementation. Usability testing sessions allowed for real-time observations of physician interactions with the EHR and clarified the need to adopt CDS implementation to match physicians’ varied workflows. Physicians had meaningful feedback on ideal content, display, information organization, and technical barriers to limit cognitive burden. Collected data informed CDS design changes which should increase end user satisfaction, widespread CDS adoption, and improved patient outcomes.

### Limitations

This study has limitations. Physicians interviewed were in practice for a mean of 15 years and themes related to usability and users’ experiences may not be generalizable to physicians with less clinical experience. Early-career physicians may find CDS clinical content and recommendations more useful than other physicians, might be more willing to use risk prediction tools, and may identify fewer barriers to implementation. Physicians in our study had previous access to other CDS tools to assist in decision-making for other common ED presentations (eg, atrial fibrillation, pulmonary embolism) which may influence their perspectives.^45,46^ Physicians from EDs with no previous exposure to CDS may report more and/or differing barriers to CDS use due to nonfamiliarity with health care technology. In addition, the clinical practice environment (lower overall admission rates, high outpatient follow-up rates, high percentage of patients with a primary care physician) may limit generalizability of CDS recommendations to other settings. Additionally, our electronic physician survey was completed by many physicians who had not used the tool in clinical practice (39%) which may limit interpretation.

## Conclusions

This mixed-methods qualitative study of ED physicians who used a CDS in clinical practice identified several key usability factors as well as key barriers and facilitators to implementation. Physicians valued the risk estimates, collated clinical data, and medication management that CDS provided and believed that CDS use would improve outcomes for patients with HF. Findings were incorporated into CDS redesign and will be used to guide systemwide implementation. Future studies of usability and implementation factors are needed to maximize physician use and clinical effect.

## Notes

Supplement 1. **eFigure 1.** Original CDS Used in the Pilot Study **eFigure 2.** Semi-Structured Interview Guide **eFigure 3.** Codebook Used for Qualitative Analysis **eTable.** Methods Used to Ensure Qualitative Rigor **eFigure 4.** Intersection of Theoretical Frameworks Used in Thematic Analysis of ED Physician Interviews and Usability Testing Sessions

Supplement 2.
Data Sharing Statement
