# Supplementary material for: Physicians’ Perceptions of Clinical Decision Support to Treat Patients With Heart Failure in the ED
Source: JAMA Netw Open. 2023 Nov 21;6(11):e2344393. doi: 10.1001/jamanetworkopen.2023.44393 (PMC10663967; doi:10.1001/jamanetworkopen.2023.44393)
Supplement: Supplement 1. — eFigure 1. Original CDS Used in the Pilot Study eFigure 2. Semi-Structured Interview Guide eFigure 3. Codebook Used for Qualitative Analysis eTable. Methods Used to Ensure Qualitative Rigor eFigure 4. Intersection of Theoretical Frameworks Used in Thematic Analysis of ED Physician Interviews and Usability Testing Sessions [file jamanetwopen-e2344393-s001.pdf]

# Supplemental Online Content

Casey SD, Reed ME, LeMaster C, et al. Physicians' perceptions of clinical decision support to treat patients with heart failure in the ED. *JAMA Netw Open.* 2023;6(11):e2344393. doi:10.1001/jamanetworkopen.2023.44393

**eFigure 1.** Original CDS Used in the Pilot Study

**eFigure 2.** Semi-Structured Interview Guide

**eFigure 3.** Codebook Used for Qualitative Analysis

**eTable.** Methods Used to Ensure Qualitative Rigor

**eFigure 4.** Intersection of Theoretical Frameworks Used in Thematic Analysis of ED Physician Interviews and Usability Testing Sessions

This supplemental material has been provided by the authors to give readers additional information about their work.

.

© 2023 Casey SD et al. *JAMA Network Open.*

**eFigure 1.** Displayed CDS information for a sample patient.

Heart Failure Report

ACUTE HEART FAILURE REPORT: LOWER RISK

**The calculated Acute Heart Failure risk today is lower (approximately 2-3% expected 30-day mortality)**

Your patient's calculated 30-day mortality risk is lower than most patients we routinely discharge from the ED. *Historically the average 30-day mortality rate in all KPNC ED patients with Acute Heart Failure is 4%.*

Consider ED Discharge if all of the following are true:

- No additional high-risk features: a) new heart failure with reduced ejection fraction, b) severe valvular disease, c) atrial fibrillation with persistent RVR, d) concomitant infection, e) worsening kidney disease (Cr 0.5 mg/dL over baseline), or f) social limitations impacting outpatient care plan.
- Symptomatically improving 2-3 hours after initial treatment
- Safe follow up plan is arranged.

Consider HBS or CDA Consult if any high-risk features are present, or more diuresis is needed (patients >6 lbs above dry weight and/or still symptomatic after treatment.

The Acute Heart Failure risk score is calculated using over 60 variables. The score was developed and validated in over 18,000 ED patients in KP Northern California. To review the study and for more information, click here.

## Weight Readings

| | 7/1/2023 0223 | 7/4/2023 2115 |
|---|---|---|
| Weight: | 133.4 kg (294 lb) | 132 kg (291 lb 0.1 oz) |
| Scale Type: | BED | BED |

## Brief Vitals (last day)

| Date/Time | Temp | Pulse | Resp | BP | FiO2 (%) | O2 Delivery | O2 (LPM) | SpO2 | Weight | Who |
|---|---|---|---|---|---|---|---|---|---|---|
| 07/05/23 1301 | 97.8 °F (36.6 °C) | 106 | 20 | 96/72 | — | NC | 3 LPM | 91 % | — | AC |
| 07/05/23 1200 | — | 106 | 22 | 94/67 | — | NC | 3 LPM | 94 % | — | VC |
| 07/05/23 1005 | — | 118 | 22 | 98/72 | — | NC | 4 LPM | 86 % | — | AC |
| 07/05/23 0826 | 97.7 °F (36.5 °C) | 104 | 20 | 113/81 | — | NC | 4 LPM | 92 % | — | AC |
| 07/05/23 0800 | — | 97 | — | 106/73 | — | NC | 4 LPM | 96 % | — | VC |
| 07/05/23 0600 | — | 98 | — | 94/72 | — | NC | 4 LPM | 95 % | — | VC |
| 07/05/23 0400 | — | 99 | 26 | 93/66 | — | NC | 4 LPM | 96 % | — | VC |
| 07/05/23 0300 | — | 105 | 24 | 94/78 | — | NC | 4 LPM | 93 % | — | RF |
| 07/05/23 0215 | — | 102 | 22 | 98/71 | — | NC | 93 LPM | 90 % | — | RF |
| 07/05/23 0000 | — | 108 | 20 | 101/73 | — | NC | 4 LPM | 92 % | — | RF |
| 07/04/23 2300 | — | 119 | 23 | 111/64 | — | NC | — | 93 % | — | RF |
| 07/04/23 2157 | — | 120 | 26 | 109/70 | — | NC | 4 LPM | 100 % | — | RF |
| 07/04/23 2146 | — | 119 | 24 | — | — | — | — | 93 % | — | RF |
| 07/04/23 2130 | — | 126 | 25 | 124/83 | — | NC | 4 LPM | 93 % | — | RF |
| 07/04/23 2115 | — | — | — | — | — | — | — | — | 132 kg (291 lb 0.1 oz) | AV |
| 07/04/23 2048 | — | 120 | 40 | 135/78 | — | NC | 4 LPM | 88 % | — | UD |

## Cardiac Rhythm (last day)

| Date/Time | Monitored Cardiac Rhythm | Who |
|---|---|---|
| 07/05/23 1301 | NSR-Normal Sinus Rhythm | AC |
| 07/05/23 0836 | NSR-Normal Sinus Rhythm | AC |
| 07/05/23 0800 | NSR-Normal Sinus Rhythm | VC |
| 07/04/23 2130 | ST-Sinus Tachycardia | RF |

## Cardiac Studies (Last 5 Years)

| Date | Order Name | Status | Specimen ID |
|---|---|---|---|
| 5/9/2023 3:31 PM | CARDIAC DEVICE CHECK, REMOTE, CV | Final | 11805066414 |

© 2023 Casey SD et al. *JAMA Network Open.*

| | | | |
|---|---|---|---|
| 8/3/2022 9:10 AM | ECHOCARDIOGRAM, TRANSTHORACIC, TRANSESOPHAGEAL AND STRESS | Final | 050001913337192 |
| 11/4/2020 6:45 AM | DEVICE/ELECTROPHYSIOLOGY PROCEDURE | Final | 020001060084018 |

## EKG Results

| Date | Order Name | Sensitivity | Status | Description | Specimen ID | Source |
|---|---|---|---|---|---|---|
| 7/5/2023 10:21 AM | EKG 12 OR MORE LEADS W INT & RPT | | Preliminary | | MUSENCA14650013 | |
| 7/3/2023 3:05 PM | EKG 12 OR MORE LEADS W INT & RPT | | Final | | MUSENCA14646902 | |
| 6/18/2023 10:24 AM | EKG 12 OR MORE LEADS W INT & RPT | | Final | | MUSENCA14607874 | |
| 6/13/2023 6:48 PM | EKG 12 OR MORE LEADS W INT & RPT | | Final | | MUSENCA14596741 | |
| 1/22/2023 8:05 PM | EKG 12 OR MORE LEADS W INT & RPT | | Final | | MUSENCA14213291 | |
| 1/4/2023 5:21 PM | EKG 12 OR MORE LEADS W INT & RPT | | Final | | MUSENCA14165004 | |
| 12/29/2022 8:23 PM | EKG 12 OR MORE LEADS W INT & RPT | | Final | | MUSENCA14149684 | |
| 12/14/2022 10:54 AM | EKG 12 OR MORE LEADS W INT & RPT | | | | | |
| 12/13/2022 3:26 PM | TRANSTHORACIC ECHO (TTE) COMPLETE CARD, CV | | Final | | 11804262975 | |
| 12/13/2022 3:25 PM | EKG 12 OR MORE LEADS W INT & RPT | | | | | |
| 12/13/2022 1:21 PM | EKG 12 OR MORE LEADS W INT & RPT | | | | | |
| 12/13/2022 12:11 PM | EKG 12 OR MORE LEADS W INT & RPT | | Final | | MUSENCA14109550 | |

## Recent Lab Studies

| | 1/25/2023 12:27 PM | 1/18/2023 11:42 AM | 1/18/2023 11:33 AM | 1/17/2023 10:41 AM | 1/9/2023 11:18 AM | 1/5/2023 10:23 AM | 12/29/2022 2:02 PM | 12/22/2022 10:37 AM | 12/20/2022 12:18 PM | 12/18/2022 11:25 AM | 12/18/2022 6:13 AM | 12/17/2022 3:44 AM | 12/16/2022 3:49 PM | 12/16/2022 10:22 AM | 10 12 |
|---|---|---|---|---|---|---|---|---|---|---|---|---|---|---|---|
| TROP | — | — | — | — | — | — | — | — | — | 20 ^ | — | — | 25 ^ | — | — |
| BNP | — | — | — | — | — | — | — | — | — | — | — | 1,582 ^ | — | 1,399 ^ | — |
| K | 5.3 | 4.9 | 4.6 | 6.3 !! | 4.6 | 5.5 ^ | 5.4 ^ | 5.1 | 5.5 ^ | — | 4.1 | 3.4 v | 3.8 | 4.5 | — |
| CR | 0.90 | — | 0.80 | 1.25 ^ | 0.85 | 0.96 | 0.93 | 0.82 | 1.18 ^ | — | 0.93 | 0.62 | 0.67 | 0.86 | 0.7 |

## Recent ED/IP Visits

| | | |
|---|---|---|
| 2 days ago | RCHLAB | HEART FAILURE W REDUCED LVEF 41-49%, UNSPECIFIED ACUITY ..., Lab |
| 1 week ago | | ABNL LABORATORY FINDING ..., ED (Discharge) |
| 1 week ago | RCHLAB | HEART FAILURE W REDUCED LVEF 41-49%, UNSPECIFIED ACUITY ..., Lab |
| 2 weeks ago | RCHLAB | HEART FAILURE W REDUCED LVEF 41-49%, UNSPECIFIED ACUITY ..., Lab |

© 2023 Casey SD et al. *JAMA Network Open.*

## OUTPATIENT CARDIAC MEDICATIONS

| | Disp | Start | End |
|---|---|---|---|
| ACE Inhibitors | | | |
| **Lisinopril (PRINIVIL/ZESTRIL) 20 mg Oral Tab**<br>Sig - Route: Take 20 mg by mouth daily - Oral<br>Class: Historical Med | | | |
| Diuretic - Thiazides and Related | | | |
| **hydroCHLOROthiazide (ESIDRIX/HYDRODIURIL) 25 mg Oral Tab**<br>Sig - Route: Take 1 tablet by mouth daily - Oral<br>Class: zRefill-Pharmacy Use Only | 100 tablet | 3/6/2023 | 3/5/2025 |
| Calcium Channel Blockers - Dihydropyridines | | | |
| **amLODIPine (NORVASC) 10 mg Oral Tab**<br>Sig - Route: Take 1 tablet by mouth daily - Oral<br>Patient not taking: Reported on 4/13/2023<br>Class: File | 100 tablet | 6/30/2022 | 6/29/2024 |
| Calcium Channel Blockers - Benzothiazepines | | | |
| **dilTIAZem (TIAZAC) 180 mg Oral 24hr SA Cap**<br>Sig - Route: Take 360 mg by mouth daily - Oral<br>Class: Historical Med | | 4/11/2023 | 6/10/2023 |
| Beta Blockers Cardiac Selective | | | |
| **Metoprolol Succinate (TOPROL XL) 100 mg Oral 24hr SR Tab**<br>Sig - Route: Take 100 mg by mouth daily - Oral<br>Class: Historical Med | | 4/11/2023 | 4/10/2024 |

## OUTPATIENT LOOP DIURETIC MEDICATIONS

| | Disp | Start | End |
|---|---|---|---|
| Diuretic - Loop | | | |
| **Furosemide (LASIX) 20 mg Oral Tab**<br>Sig - Route: Take 20 mg by mouth daily - Oral<br>Class: Historical Med | | 4/11/2023 | 4/10/2024 |

**Clinical recommendations for ED diuresis:**

- For diuretic-naïve patients, start with 40mg IV Lasix.
- For patients on a diuretic, consider doubling their bioequivalent oral outpatient dose (see table below). For example, for patients on 40mg po bid Lasix, start with 40mg IV Lasix. (IV Lasix is double the potency of PO Lasix)
- If initial dosing does not produce a diuresis in the first 1-2 hours, consider repeating with a higher dose.

| | Furosemide | Torsemide | Bumetanide |
|---|---|---|---|
| Relative intravenous potency (mg) | 40 | 20 | 1 |
| Oral : intravenous dosing | 1 : 2 | 1 : 1 | 1 : 1 |
| Bioavailability (%) | 10–100 | 80–100 | 80–100 |
| Drug half-life (h) | 1.5–2.0 | 3–4 | 1.0–1.5 |
| Duration of effect (h) | 6–8 | 6–16 | 4–6 |

**OUTPATIENT Management Recommendations:**

For patients with an Ejection Fraction less than 45%, **Guideline Directed Medical Therapy** includes:

1) Either an ACE-Inhibitor (ACEI), Angiotensin Receptor Blocker (ARB), or an Angiotensin Receptor/Neprilysin Inhibitor (ARNi) medication
2) Beta Blocker (e.g. Coreg, Bisoprolol, Metoprolol)
3) Mineralocorticoid receptor antagonist (e.g. Spironolactone)
4) A Sodium-Glucose Cotransporter 2 inhibitor medication (SGLT2i) e.g. Jardiance

**Recommendation:** If patient is not on all of these medications, consider starting prior to discharge and route note to patient's cardiologist or PCP as applicable.

- If not on Beta Blocker, start low dose (e.g. 3.125 mg bid Coreg)
- If not on ACEI/ARB, start low dose (e.g. Losartan 25mg qd) and order 3-4 day K and Cr lab follow up (route order to outpatient cardiologist)
- If not on Jardiance, start at ½ tab of 25mg (12.5mg qd)
- If not on Spironolactone, start low dose (12.5mg qd) and order 3–4 day outpatient Cr and K follow up (route order to outpatient cardiologist). Defer starting if increased Cr in ED.

**Discharge Recommendations**

- Please ensure all patients with EF < 45% enrolled in **CCM HF program** (eConsult)
- If patient not back to dry weight, consider adding a note in their diuretic discharge order to double their usual diuretic dose for 3 days, then go back to their baseline dose.
- AVS: Please include (copy and paste) the "Stoplight" or "Heart Failure Zone Tool" into the AVS for home care.
- Please route your note to the patient's cardiologist and/or PCP as applicable. When applicable also include their Heart Failure Transitions Nurse.

© 2023 Casey SD et al. *JAMA Network Open.*

**eFigure 2.** Semi-structured interview guide.

We are interviewing physicians about their experiences using clinical decision support for patients in the emergency department for an acute exacerbation of heart failure. Your responses will help us improve the clinical decision support we provide.

We are going to ask you to assess a patient with heart failure. Please "think aloud" during the case and share with us what you are thinking in real time as you work through it.

Please imagine that you have been assigned a patient who is here with shortness of breath. Before you go see them, what is your typical workflow in finding out more about this patient?

- What steps do you take to gather information from the EHR?

You perform a physical exam. The patient has bilateral rales and pretibial edema. You return to your workstation to place orders.

- Can you describe your workflow from this point?
- Have you used the heart failure clinical decision support tool?
- Can you tell me where you would find the prompt that would allow you to access the tool?

Can you please access the HF clinical decision support tool and tell us how this may or may not be useful in this patient's diagnosis, treatment, and determination of final disposition?

- Was the displayed clinical content useful? Why or why not?
- What might be some barriers and facilitators of using the CDS where you practice?
- Do you think the location of the prompt was effective? Why or why not?
- How did the CDS help you make decisions? How did it make you feel when making those decisions?
- Did the CDS complement or complicate your clinical workflow?
- Did the CDS integrate into your workflow in admitting a patient with HF?
- Did the CDS integrate into your workflow in discharging a patient with HF?

The risk estimates provided by the tool are derived from a process that you may not completely understand.

- How do you feel about using technology such as this to assist you in your medical decision making?

© 2023 Casey SD et al. *JAMA Network Open.*

**eFigure 3.** Codebook used for qualitative analysis

A. Internal policies
    1. Expectations v. capacity
        i. Workload
        ii. Scope and responsibility
B. Human/computer interface
    1. User control and freedom
        i. Dyssynchronous use
            1. Optimal timing to display prompt
        ii. Lack of customizability
            1. Optimal placement of prompt
            2. Customizable presentation of clinical information
    2. Flexibility and Efficiency
        i. Faster disposition
    3. Aesthetic and minimalist design
        i. Information saturation
        ii. Indistinct color
    4. Recognition v. recall
        i. Cannot order medications
        ii. Communication between CDS and EHR ordering
C. Workflow
    1. Collation of information
    2. Efficiency
        i. Utility for hospitalizing a patient
        ii. Utility for discharging a patient home

© 2023 Casey SD et al. *JAMA Network Open.*

D. People

   1. Trust in research team

   2. Trust in emergency department leadership

E. Clinical content

   1. Utility of quantitative risk date

      i. Shared decision making

   2. Oversaturation of clinical data

      i. Clinically useful data to include

   3. Collation of data

© 2023 Casey SD et al. *JAMA Network Open.*

**eTable**. Methods used to ensure qualitative rigor.

| | Purpose | Strategies applied in our study to achieve rigor |
|---|---|---|
| **Credibility** | Establishes validity | Interview duration was approximately 60 minutes providing for lengthy and intense contact with physicians. Three practicing ED physicians conducted interviews along with one non-physician consultant who had training in design strategy. We triangulated sources of our generated themes. We examined transcripts to identify quotes that went against each of our major themes. |
| **Dependability** | Ensures consistent application of the study protocol | We created a draft of the study protocol. Two practicing emergency physicians independently coded interview data. Discrepancies were discussed between coding physicians to achieve consensus. |
| **Confirmability** | Ensures that results will be replicable | Our study team consisted of a diverse group of physicians and non-physicians. We also reported demographics of those involved in interviews and coding in the manuscript. We routinely reflected on our own professional experiences and discussed how these may influence interpretations of the data during the study. |
| **Transferability** | Assesses the generalizability of our results to other health care settings | Codes, themes, and narrative interpretations were derived from thick descriptive data provided by our interview transcripts. We used a semi-structured interview format to probe interview questions defined *a priori* while allowing for new themes to develop |

© 2023 Casey SD et al. *JAMA Network Open.*

**eFigure 4.** Intersection of theoretical frameworks used in thematic analysis of ED physician interviews and usability testing sessions.

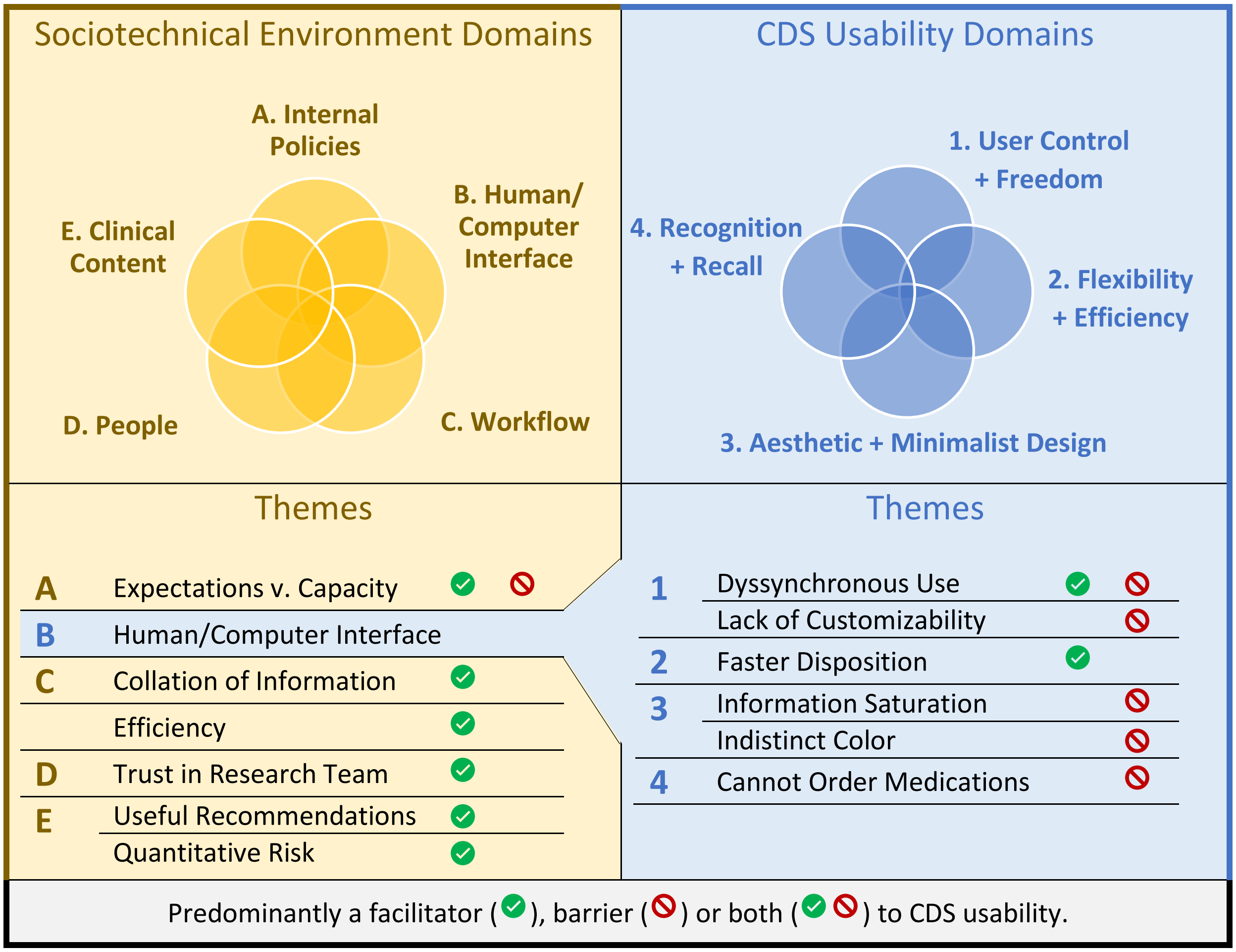


© 2023 Casey SD et al. *JAMA Network Open.*
