# Supplementary material for: Physicians’ Perceptions of Clinical Decision Support to Treat Patients With Heart Failure in the ED
Source: JAMA Netw Open. 2023 Nov 21;6(11):e2344393. doi: 10.1001/jamanetworkopen.2023.44393 (PMC10663967; doi:10.1001/jamanetworkopen.2023.44393)
Supplement: Supplement 2. — Data Sharing Statement [file jamanetwopen-e2344393-s002.pdf]

Data Sharing Statement

Casey. Physicians' Perceptions of Clinical Decision Support to Treat Patients With Heart Failure in the ED. *JAMA Netw Open*. Published November 21, 2023. doi:10.1001/jamanetworkopen.2023.44393

Data
Data available: Yes
Data types: Deidentified participant data
How to access data: scott.d.casey@kp.org
When available: With publication

Supporting Documents
Document types: None

Additional Information
Who can access the data: Anyone requesting the data
Types of analyses: Qualitative data will be made available to anyone requesting the data for any type of analysis provided that the request is approved by internal data sharing agreements.
Mechanisms of data availability: After approval of proposal
Any additional restrictions: none
